# Clinical Assessment of Pediatric Patients with Differentiated Thyroid Carcinoma: A 30-Year Experience at a Single Institution

**DOI:** 10.1007/s00268-020-05598-9

**Published:** 2020-05-21

**Authors:** Kwangsoon Kim, Cho Rok Lee, Sang-Wook Kang, Jandee Lee, Jong Ju Jeong, Kee-Hyun Nam, Woong Youn Chung

**Affiliations:** 1 Department of Surgery, College of Medicine, The Catholic University of Korea, Seoul, Korea; 2 Department of Surgery, Yonsei University College of Medicine, Seoul, 03722 Korea

## Abstract

**Background:**

Thyroidectomy is the typical treatment for pediatric thyroid carcinoma; total thyroidectomy is commonly performed. We aimed to report our experience at a single tertiary institution and to evaluate the risk factors for recurrence, especially based on surgical extent, in pediatric patients with differentiated thyroid carcinoma (DTC).

**Methods:**

A data of 94 pediatric patients who underwent thyroid surgery for DTC from January 1982 to December 2012 at Yonsei University Hospital (Seoul, Korea) were reviewed. The clinicopathologic features and surgical outcomes were retrospectively analyzed through complete chart reviews.

**Results:**

The mean age was 16.6 ± 3.0 (range, 5–19) years. Fourteen patients had recurrence. Tumor size >2 cm (hazard ratio [HR], 14.241; *p* = 0.011) and positive lymph nodes (HR, 1.056; *p* = 0.039) were significant risk factors for disease-free survival (DFS) in multivariate analysis. In Kaplan–Meier analysis, a statistically significant difference was noted in the DFS according to tumor size 2 cm (*p* < 0.001). However, the DFS was not significantly different between the bilateral total thyroidectomy (BTT) and less than BTT groups (*p* = 0.215).

**Conclusions:**

BTT remains the treatment of choice in pediatric patients with DTC. Lobectomy may be considered for patients with limited disease, including those with tumor size <2 cm, no suspicious lymph nodes, intrathyroidal lesion, and no multifocal disease.

## Introduction

Thyroid carcinoma is very rare during childhood and accounts for 1.8% of all thyroid malignancies, according to the surveillance, epidemiology, and end results program [1]. Among thyroid carcinomas, differentiated thyroid carcinoma (DTC) is the most common endocrine carcinoma in pediatric patients, accounting for 90–95% of all pediatric thyroid carcinomas [2, 3]. Papillary thyroid carcinoma (PTC) accounts for >90% of all cases of DTC, and follicular thyroid carcinoma is a rare histological type [4, 5].

Pediatric DTC can be differentiated from adult DTC based on several characteristics. The disease is more extensive, with more common extrathyroidal extension (ETE) and a higher risk of recurrence, in pediatric patients [6–8]. Several studies have reported that the incidence of distant metastasis is up to 25% and that of lymph node metastasis ranges from 40 to 80% [8–10]. Despite the more aggressive disease features and higher risk of recurrence, the long-term outcome has been reported to be better in pediatric patients than in adult patients [11, 12].

Treatment of pediatric DTC generally involves surgery and postoperative treatment, such as radioactive iodine (RAI) therapy and thyroid-stimulating hormone suppression. Surgery is the main approach for treating DTC, and the extent of surgery ranges from lobectomy to bilateral total thyroidectomy (BTT). According to the American Thyroid Association (ATA) management guidelines, the treatment of choice for pediatric patients with DTC is thyroidectomy, especially BTT [13]. BTT is preferred because of the increased incidence of bilateral and multifocal disease, as well as an increased risk of recurrence [6, 9, 14]. In addition, RAI therapy is administered and thyroglobulin (Tg) serves as a tumor marker when BTT is performed [14, 15]. However, the disadvantages of BTT could lead to various complications, such as transient/persistent postoperative hypoparathyroidism and recurrent laryngeal nerve injury [16, 17]. Permanent hypoparathyroidism may develop in <2.5% of the patients, and permanent recurrent laryngeal nerve palsy may develop in approximately 1% of the patients, even when the procedure is performed by experienced surgeons [18, 19].

This retrospective study aimed to report our experience at a single tertiary institution and to evaluate the risk factors for recurrence, especially based on the extent of surgery, in pediatric patients with DTC.

## Methods

### Patients

The data of 110 pediatric (age ≤19 years) patients with DTC who underwent thyroidectomy at Yonsei University Hospital (Seoul, Korea) between January 1982 and December 2012 were retrospectively reviewed. Sixteen pediatric patients were excluded because they were lost to follow-up. The data of 94 patients were completely analyzed by reviewing the medical charts and pathology reports. Among them, 61 (64.9%) patients underwent BTT and/or modified radical neck dissection (mRND) and 33 (35.1%) patients underwent less than BTT. The mean follow-up duration was 148.6 ± 81.6 months (range, 60–452 months). This study was approved by the local institutional review board (approval no. 4-2017-1099), which waived the requirement for informed consent due to the retrospective nature of this study.

### Surgical treatment

Pediatric patients with DTC were followed up based on the ATA management guidelines for children [13]. According to the ATA guidelines, BTT is recommended for most children owing to an increased incidence of bilaterality and multifocal disease in this population. In long-term analysis, BTT has been shown to decrease the risk of recurrence [20]. However, for the study patients, lobectomy was performed only when the disease was apparently limited, such as for an intrathyroidal lesion or when bilaterality was not observed on preoperative evaluation. Prophylactic central compartment node dissection was regularly performed for all pediatric patients. Therapeutic mRND was performed in cases with clinically suspicious or pathologically confirmed N1b disease.

### Postoperative management and follow-up

All pediatric patients were managed after surgical treatment according to the ATA management guidelines for children [13]. All patients took suppressive doses of L-thyroxine and were regularly followed up with physical examination, thyroid function testing, assessment of anti-Tg antibody concentrations, and neck ultrasonography every 3–6 months, and annually thereafter. RAI ablation was performed at 6–8 weeks postoperatively, and whole-body scans were performed at 5–7 days after RAI ablation in patients who underwent BTT. If necessary, additional diagnostic imaging, such as computed tomography, positron emission tomography/computed tomography, and/or RAI whole-body scanning were performed to confirm recurrent disease.

### Statistical analysis

All statistical analyses were performed with the SPSS software package (SPSS version 23.0 for Windows; SPSS, Chicago, IL, USA). Continuous variables are reported as mean with standard deviation, and categorical variables are reported as number with percentage. Student’s *t*-test, Chi-square test, or Fisher’s exact test, if necessary, was used to compare the two groups. To determine the optimal cutoff value of the lymph node ratio (LNR), receiver operating characteristic (ROC) curve analysis was performed. Univariate and multivariate Cox regression analyses were performed to validate the predictors of disease-free survival (DFS). Hazard ratios (HRs) with 95% confidence intervals (CIs) were calculated. Kaplan–Meier survival analysis with log-rank test was performed to compare the DFS among the different groups. A statistically significant difference was defined as *p* < 0.05.

## Results

### Baseline clinicopathologic characteristics

Table 1 presents the baseline clinicopathologic characteristics of the 94 total pediatric patients who underwent thyroid surgery for DTC. The mean age of the patients was 16.6 years (range, 5–19 years). Six (6.4%) patients were aged ≤10 years, and there were 84 (89.4%) female patients. The mean tumor size was 2.1 cm (range, 0.3–9 cm), and most tumors (92.6%) were diagnosed as PTC. Multifocality, bilaterality, and ETE of the disease were diagnosed in 22 (23.4%), 14 (14.9%), and 34 (36.2%) patients, respectively. The tumor-node-metastasis (TNM) stage was classified based on the 8th edition of the American Joint Committee on Cancer/Union for International Cancer Control TNM staging system. The distribution of patients according to each T stage was as follows: stage 1, 42 patients (44.7%); stage 2, 17 patients (18.1%); stage 3, 31 patients (33.0%); and stage 4, 4 patients (4.3%). The number of patients diagnosed with N1a and N1b was 41 (43.6%) and 30 (31.9%), respectively. Only two patients (2.1%) had distant metastasis in the lung at the time of the thyroid carcinoma diagnosis. Fourteen (14.9%) patients were diagnosed with recurrence after the initial treatment.

**Table 1:** Baseline clinicopathologic characteristics

Total 94 patients	
Age (years)	16.6 ± 3.0 (range, 5–19)
≤10	6 (6.4%)
>10	88 (93.5%)
Male: female	1: 8.4 (10: 84)
Tumor size (cm)	2.1 ± 1.4 (range, 0.3–9)
Type of carcinoma	
PTC	87 (92.6%)
FTC	7 (7.4%)
Multifocality	22 (23.4%)
Bilaterality	14 (14.9%)
ETE	34 (36.2%)
Extent of operation	
Less than BTT	33 (35.1%)
BTT and/or mRND	61 (64.9%)
Harvested LNs	21.0 ± 24.0
Positive LNs	6.5 ± 8.6
T stage	
T1	42 (44.7%)
T2	17 (18.1%)
T3	31 (33.0%)
T4	4 (4.3%)
N stage	
N0	23 (24.5%)
N1a	41 (43.6%)
N1b	30 (31.9%)
M stage	
M1	2 (2.1%)
Recurrence	14 (14.9%)
Follow up duration (months)	148.6 ± 81.6 (range, 60–452)

Data are expressed as the patient number (%) or mean ± SD

*PTC* papillary thyroid carcinoma, *FTC* follicular thyroid carcinoma, *ETE* extrathyroidal extension, *BTT* bilateral total thyroidectomy, *mRND* modified radical neck dissection, *LN* lymph node, *T* tumor, *N* node, *M* metastasis

### Comparison of baseline clinicopathologic characteristics according to tumor size, age and LNR

The patients were divided into the following two groups: small tumor group (≤2 cm, *n* = 50, 53.2%) and large tumor group (>2 cm, *n* = 44, 46.8%). There were no statistically significant differences in age, proportion of female patients, multifocality, and bilaterality between the two groups. However, the large tumor group underwent a significantly more extensive surgery than the small tumor group (77.3% vs. 54.0%, *p* = 0.030). The large tumor group presented a significantly higher ETE rate, number of harvested lymph nodes, and number of positive lymph nodes than the small tumor group (*p* < 0.001, *p* = 0.005, and *p* = 0.012, respectively). On the basis of the T and N stages, the large tumor group had a significantly higher grade (*p* < 0.001 and *p* = 0.030, respectively). The recurrence rates were also significantly higher in the large tumor group than in the small tumor group (2.0% vs. 29.5%, *p* < 0.001; Table 2).

**Table 2:** Comparison of baseline clinicopathological characteristics according to tumor size (2 cm)

	Tumor size ≤2 cm (*n* = 50)	Tumor size >2 cm (*n* = 44)	*p* value
Age (years)	16.9 ± 3.0	16.4 ± 3.0	0.426
Female	46 (92.0%)	38 (86.4%)	0.507
Extent of operation			0.030
Less than BTT	23 (46.0%)	10 (22.7%)	
BTT and/or mRND	27 (54.0%)	34 (77.3%)	
Tumor size (cm)	1.1 ± 0.5	3.2 ± 1.2	<0.001
Multifocality	11 (22.0%)	11 (25.0%)	0.809
Bilaterality	5 (10.0%)	9 (20.5%)	0.245
ETE	8 (16.0%)	26 (59.1%)	<0.001
Harvested LNs	14.5 ± 19.2	28.3 ± 26.8	0.005
Positive LNs	4.4 ± 7.2	8.9 ± 9.5	0.012
T stage			<0.001
T1	42 (84.0%)	0	
T2	0	17 (38.6%)	
T3	8 (16.0%)	23 (52.3%)	
T4	0	4 (9.1%)	
N stage			0.030
N0	14 (28.0%)	9 (20.5%)	
N1a	26 (52.0%)	15 (34.1%)	
N1b	10 (20.0%)	20 (45.4%)	
Recurrence	1 (2.0%)	13 (29.5%)	<0.001

Data are expressed as the patient number (%) or mean ± SD

A statistically significant difference was defined as *p* < 0.05

*BTT* bilateral total thyroidectomy, *mRND* modified radical neck dissection, *ETE* extrathyroidal extension, *LN* lymph node, *T* tumor, *N* node

Table 3 shows the comparison of the baseline clinicopathologic characteristics according to age. The patients were divided into the following two groups according to age 17 years because the mean age of the study patients was 16.6 years: younger pediatric group (≤17 years, *n* = 35, 37.2%) and older pediatric group (>17 years, *n* = 59, 62.8%). There were no statistically significant differences in the proportion of female patients, mean tumor size, multifocality, bilaterality, T stage, and recurrence rate between the two groups. However, the younger pediatric group underwent a significantly more extensive surgery than the older pediatric group (82.9% vs. 54.2%, *p* = 0.007). The younger pediatric group presented a significantly higher ETE rate, number of harvested lymph nodes, and number of positive lymph nodes than the older pediatric group (*p* < 0.026, *p* = 0.002, and *p* = 0.020, respectively). On the basis of the N stage, the younger pediatric group had a significantly higher grade (*p* = 0.027).

**Table 3:** Comparison of baseline clinicopathological characteristics according to age (17 years)

	Age ≤17 years (*n* = 35)	Age >17 years (*n* = 59)	*p* value
Age (years)	13.7 ± 3.1	18.4 ± 0.8	<0.001
Female	29 (82.9%)	55 (93.2%)	0.166
Extent of operation			0.007
Less than BTT	6 (17.1%)	27 (45.8%)	
BTT and/or mRND	29 (82.9%)	32 (54.2%)	
Tumor size (cm)	2.3 ± 1.0	1.9 ± 1.5	0.225
Multifocality	9 (25.7%)	13 (22.0%)	0.802
Bilaterality	7 (20.0%)	7 (11.9%)	0.371
ETE	18 (51.4%)	16 (27.1%)	0.026
Harvested LNs	30.7 ± 29.9	15.2 ± 17.5	0.002
Positive LNs	9.2 ± 10.4	4.9 ± 6.9	0.020
T stage			0.059
T1	10 (28.6%)	32 (54.2%)	
T2	7 (20.0%)	10 (17.0%)	
T3	15 (42.9%)	16 (27.1%)	
T4	3 (8.5%)	1 (1.7%)	
N stage			0.027
N0	7 (20.0%)	16 (27.1%)	
N1a	11 (31.4%)	30 (50.9%)	
N1b	17 (48.6%)	13 (22.0%)	
Recurrence	7 (20.0%)	7 (11.9%)	0.371

Data are expressed as the patient number (%) or mean ± SD

A statistically significant difference was defined as *p* < 0.05

*BTT* bilateral total thyroidectomy, *mRND* modified radical neck dissection, *ETE* extrathyroidal extension, *LN* lymph node, *T* tumor, *N* node

The results of the comparison of the baseline clinical characteristics according to the LNR are summarized in Table 4. We evaluated the optimal cutoff value using the LNR from the ROC curve analysis, and divided the patients into the low LNR group (≤0.4, *n* = 64, 68.1%) and the high LNR group (>0.4, *n* = 30, 31.9%). There were no significant differences between the two groups except for the mean age, ETE, and N stage. The high LNR group presented a significantly higher rate of ETE (53.3% vs. 28.1%). On the basis of the N stage, the high LNR group had a significantly higher grade than the low LNR group (*p* < 0.001). However, there were no statistically significant differences in the recurrence rate between the two groups (10.9% vs. 23.3%, *p* = 0.131).

**Table 4:** Comparison of baseline clinicopathological characteristics according to lymph node ratio (0.4)

	LNR ≤0.4 (*n* = 64)	LNR >0.4 (*n* = 30)	*p* value
Age (years)	16.2 ± 3.4	17.5 ± 1.5	0.044
Female	55 (85.9%)	29 (96.7%)	0.161
Extent of operation			0.821
Less than TT	22 (34.4%)	11 (36.7%)	
TT and/or mRND	42 (65.6%)	19 (63.3%)	
Tumor size (cm)	2.0 ± 1.2	2.3 ± 1.7	0.317
Multifocality	12 (18.8%)	10 (33.3%)	0.128
Bilaterality	9 (14.1%)	5 (16.7%)	0.762
ETE	18 (28.1%)	16 (53.3%)	0.022
Harvested LNs	21.6 ± 25.3	19.5 ± 21.2	0.695
Positive LNs	4.8 ± 7.0	10.1 ± 10.6	0.005
T stage			0.061
T1	30 (46.9%)	12 (40.0%)	
T2	15 (23.4%)	2 (6.7%)	
T3	16 (25.0%)	15 (50.0%)	
T4	3 (4.7%)	1 (3.3%)	
N stage			<0.001
N0	23 (35.9%)	0	
N1a	20 (31.3%)	21 (70.0%)	
N1b	21 (32.8%)	9 (30.0%)	
Recurrence	7 (10.9%)	7 (23.3%)	0.131

Data are expressed as the patient number (%) or mean ± SD

A statistically significant difference was defined as *p* < 0.05

*LNR* lymph node ratio, *TT* bilateral total thyroidectomy, *mRND* modified radical neck dissection, *ETE* extrathyroidal extension, *LN* lymph node, *T* tumor, *N* node

### BTT and less than BTT group comparisons

The results of the comparison of the baseline clinicopathologic characteristics between the BTT and less than BTT groups are shown in Table 5. There were no statistically significant differences in the mean tumor size between the two groups (2.2 ± 1.0 vs. 1.8 ± 1.8 cm, *p* = 0.193). However, the proportion of male patients was significantly higher in the BTT group than in the less than BTT group (*p* = 0.013). The incidence of multifocality, bilaterality, and ETE was also significantly higher in the BTT group than in the less than BTT group (*p* = 0.021, *p* = 0.002, and *p* = 0.002, respectively). On the basis of the T stage, the less than BTT group had a significantly lower grade and the BTT group had a significantly higher grade (*p* = 0.002). With respect to the N stage, all patients in the less than BTT group were diagnosed with N0 or N1a stage disease, whereas N1b stage disease was diagnosed in 30 (49.2%) patients in the BTT group (*p* < 0.001). Seven (11.5%) patients in the BTT group and 7 (21.2%) patients in the less than BTT group were diagnosed with recurrence; however, this result was not statistically significant (*p* = 0.234).

**Table 5:** Comparison of baseline clinicopathological characteristics according to surgical method

	BTT and/or mRND (*n* = 61)	Less than BTT (*n* = 33)	*p* value
Age (years)	16.0 ± 3.3	17.7 ± 2.1	0.008
Female	51 (83.6%)	33 (100%)	0.013
Tumor size (cm)	2.2 ± 1.0	1.8 ± 1.8	0.193
Multifocality	19 (31.1%)	3 (9.1%)	0.021
Bilaterality	14 (23.0%)	0	0.002
ETE	29 (47.5%)	5 (15.2%)	0.002
Harvested LNs	27.5 ± 26.0	8.9 ± 13.0	<0.001
Positive LNs	8.5 ± 9.1	2.8 ± 6.3	0.002
T stage			0.002
T1	19 (31.1%)	23 (69.6%)	
T2	12 (19.7%)	5 (15.2%)	
T3	26 (42.6%)	5 (15.2%)	
T4	4 (6.6%)	0	
N stage			<0.001
N0	8 (13.1%)	15 (45.5%)	
N1a	23 (37.7%)	18 (54.5%)	
N1b	30 (49.2%)	0	
Recurrence	7 (11.5%)	7 (21.2%)	0.234

Data are expressed as the patient number (%) or mean ± SD

A statistically significant difference was defined as *p* < 0.05

*BTT* bilateral total thyroidectomy, *mRND* modified radical neck dissection, *ETE* extrathyroidal extension, *LN* lymph node, *T* tumor, *N* node

### Recurrence

Table 6 presents the approximate recurrence patterns in the patients included in this study. All patients diagnosed with recurrent disease underwent surgical treatment. Most of the patients in the BTT group, with the exception of patient no. 6, experienced recurrence at the contralateral central compartment lymph nodes. Patient no. 6 developed recurrence at the ipsilateral level 3 area and underwent mRND. In comparison, patients in the less than BTT group showed different recurrence patterns, as follows: 5 patients were confirmed to have had recurrence at the thyroid contralateral to the operated side, and patient no. 4 had recurrence at the ipsilateral surgery bed. Patient no. 1 developed recurrence bilaterally at levels 3 and 4. Patient no. 7 developed recurrence at the ipsilateral level 4 area and underwent mRND.

**Table 6:** Recurrence patterns for the study population

Patients	Age	Sex	Tumor size (cm)	Recurrence site	DFS (months)
BTT					
1	18	Female	2.7	Contralateral level 6 LNs	15
2	15	Female	3	Contralateral level 6 LNs	11
3	15	Female	3.3	Contralateral level 6 LNs	103
4	5	Female	4.8	Contralateral level 6 LNs	92
5	15	Male	4	Contralateral level 6 LNs	70
6	15	Female	2.4	Ipsilateral level 3 LNs	52
7	17	Female	2.2	Contralateral level 6 LNs	39
Less than BTT					
1	16	Female	2.8	Bilateral level 3,4 LNs	10
2	17	Female	4	Contralateral thyroid	71
3	18	Female	2.5	Contralateral thyroid	67
4	16	Female	3	Ipsilateral op bed	33
5	19	Female	2.3	Contralateral thyroid	142
6	19	Female	2.5	Contralateral thyroid	35
7	17	Female	0.8	Contralateral thyroid & ipsilateral Level 4 LNs	84

All patients who underwent BTT received radioactive iodine ablation therapy

*DFS* disease-free survival, *BTT* bilateral total thyroidectomy, *LN* lymph node

### Univariate and multivariate analyses of the risk factors for recurrence

Table 7 shows the results of the univariate and multivariate Cox regression analyses for identifying the risk factors associated with the DFS. In univariate analysis, tumor size >2 cm (HR, 17.168; *p* = 0.006), ETE (HR, 3.443; *p* = 0.027), higher T stage (T2: HR, 11.123 [*p* = 0.031]; T3: HR, 10.726 [*p* = 0.026]; T4: HR, 21.642 [*p* = 0.012]), and positive lymph nodes (HR, 1.071; *p* = 0.005) were found to be significant predictors of recurrence. Among the various risk factors, tumor size >2 cm and positive lymph nodes were identified as significant risk factors for the DFS in multivariate analysis. Especially, the most significant predictor of the DFS was tumor size >2 cm (HR, 14.241; *p* = 0.011).

**Table 7:** Univariate and multivariate analysis for disease-free survival

	Univariate		Multivariate	
	HR (95% CI)	*p* value	HR (95% CI)	*p* value
Tumor size				
≤2 cm	Ref		Ref	
>2 cm	17.168 (2.245–131.307)	0.006	14.241 (1.847–109.828)	0.011
ETE	3.443 (1.154–10.277)	0.027		
T stage				
T1	Ref			
T2	11.123 (1.241–99.686)	0.031		
T3	10.726 (1.319–87.210)	0.026		
T4	21.642 (1.962–238.774)	0.012		
Positive LNs	1.071 (1.020–1.123)	0.005	1.056 (1.003–1.111)	0.039

Data are expressed as the hazard ratio (HR) and 95% confidence interval (CI)

A statistically significant difference was defined as *p* < 0.05

*ETE* extrathyroidal extension, *LN* lymph node, *T* tumor

**Fig. 1:**
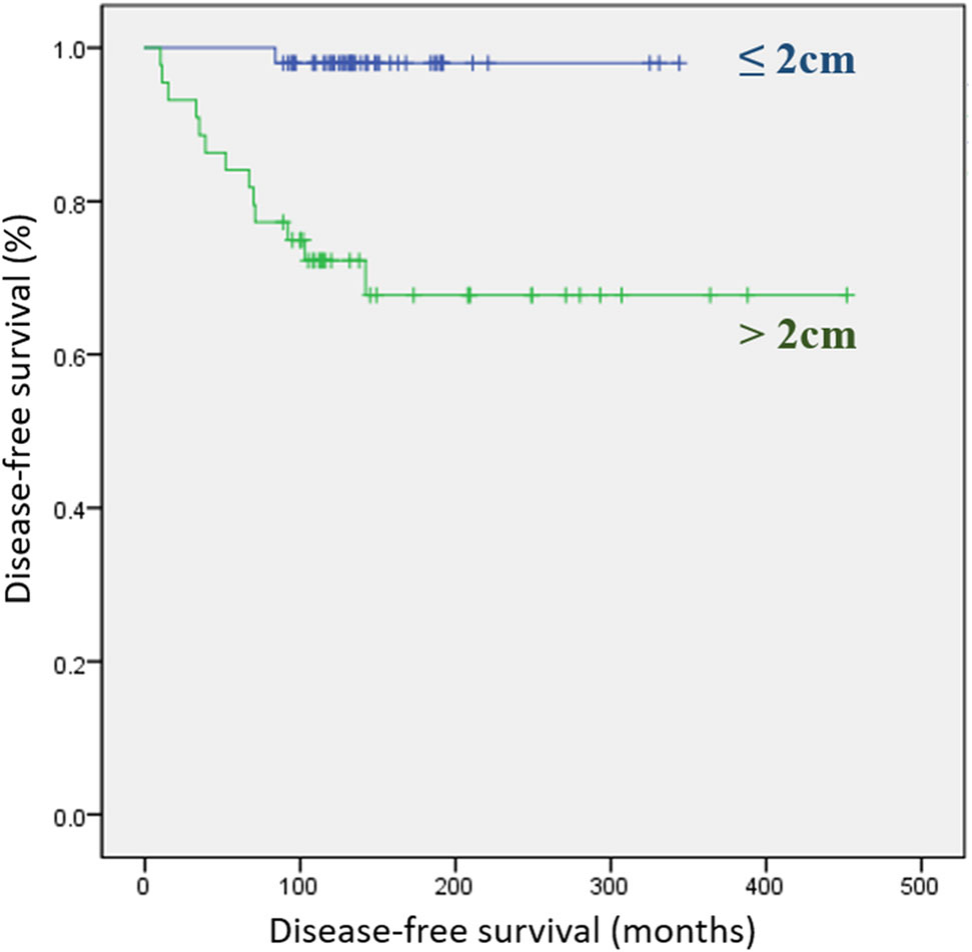
Disease-free survival curves according to tumor size (2 cm) (log-rank *p* < 0.001)

**Fig. 2:**
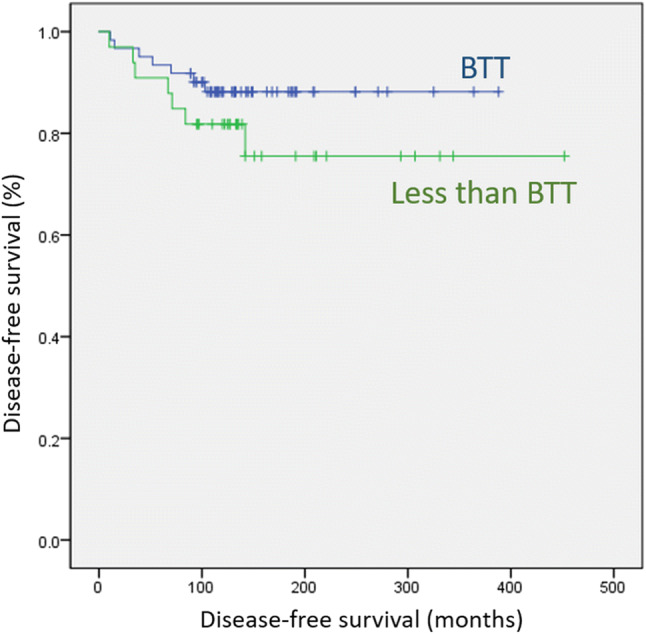
Disease-free survival curves according to extent of surgery (log-rank *p* = 0.215)

In Kaplan–Meier analysis, a statistically significant difference was noted in the DFS between the large tumor group and the small tumor group (log-rank *p* < 0.001; Fig. 1). Conversely, there was no statistically significant difference in the DFS between the BTT group and the less than BTT group (log-rank *p* = 0.215; Fig. 2).

## Discussion

The incidence of DTC has significantly increased in the last 2 decades and has also steadily increased in pediatric patients [2, 21–23]. DTC manifests more aggressive features in pediatric patients than in adults; however, the prognosis is better known in pediatric patients [6–8, 12, 24]. Similar to that in the other studies, lymph node metastasis was observed in 75.5% and ETE was observed in 36.2% of the patients in our study [6–10, 25]. According to the ATA management guidelines for children, BTT is recommended in pediatric patients with DTC because of the possibility of recurrence owing to the more extensive disease pathology in this population [13]. Other studies have also reported that BTT can reduce the incidence of recurrence compared with lobectomy [6, 9, 26]. However, BTT can cause various postoperative complications, although such complications are rare. The complications can include permanent hypoparathyroidism or vocal cord palsy, which can be associated with serious long-term problems [18, 19]. Although there is a general consensus that surgery is the primary treatment for pediatric DTC, the extent of surgery remains controversial [24]. The main issue is related to the impact of the extent of surgery on recurrence and the potential associated risk of complications. Those who favor BTT claim that the procedure is related to improved DFS without significant complications when performed by experienced surgical teams [26, 27]. However, lobectomy has been proposed to have comparable surgical outcomes in selected patients and is considerably safer than BTT [28].

In the present study, 7 (11.5%) and 7 (21.2%) patients in the BTT and less than BTT groups, respectively, experienced disease recurrence. There was a distinct pattern of recurrence in both groups. In the BTT group, all patients, except one patient, experienced recurrence in the contralateral central compartment. In comparison, 5 of the 7 patients in the less than BTT group experienced recurrence in the thyroid contralateral to the operated side. This is probably due to an invisible multifocal disease on the contralateral thyroid of the lesion at the time of DTC diagnosis. One patient who underwent lobectomy needed a reoperation within 1 month after surgery because of lateral neck node metastasis. It is possible that the lateral neck node metastasis was missed at the time of DTC diagnosis. Thus, we excluded this patient from our study population.

In this study, 1 (2.0%) and 13 (29.5%) patients in the small tumor and large tumor groups, respectively, experienced disease recurrence after the initial treatment. The recurrence rate was significantly higher in the large tumor group than in the small tumor group (*p* < 0.001), and it was also statistically significant in the Kaplan–Meier analysis (log-rank *p* < 0.001). However, there was no statistically significant difference according to a tumor size of 1 cm (log rank *p* = 0.098). Byeon et al. reported that there was no significant difference in the DFS between the papillary thyroid microcarcinoma group and the PTC >1 cm group [29]. Recently, the LNR has been suggested as a significant prognostic factor in adult patients with PTC [30]. Rubinstein et al. reported that the LNR may be a useful predictor of recurrence in pediatric patients with PTC [31]. ROC curve analysis was performed to determine the optimal cutoff LNR of 0.4. However, there was no statistically significant difference in the DFS between the low LNR and high LNR groups (log-rank *p* = 0.096).

To determine the risk factors associated with the DFS, we conducted univariate and multivariate Cox regression analyses. Only two risk factors, tumor size >2 cm and positive lymph nodes, were identified as significant risk factors for the DFS (HR, 14.241; 95% CI, 1.847–109.828 [*p* = 0.011] and HR, 1.056; 95% CI, 1.003–1.111 [*p* = 0.039], respectively). Conversely, the extent of surgery was not a risk factor for the DFS. The less than BTT group showed a high HR; however, this result was not statistically significant in the univariate analysis (HR, 2.171; 95% CI, 0.787–5.989; *p* = 0.134). Kaplan–Meier analysis for the DFS showed no significant differences between the BTT and lobectomy groups (log-rank *p* = 0.215). In contrast to the results reported in other studies, the extent of surgery did not affect recurrence in this study, which may be due to differences in the patients’ ethnicity or the patient groups in this study [4, 20, 27, 32]. Demidchik et al. suggested that lobectomy is only acceptable in limited diseases, such as small solitary intrathyroidal lesions without evidence of neck lymph node involvement [4]. Massimino et al. reported that the prognosis was favorable even after recurrence and was not related to the extent of surgery [33]. Therefore, we suggest that lobectomy may be an alternative to BTT, but only for patients with limited disease, such as those with tumor size <2 cm, no suspicious lymph nodes, intrathyroidal lesion, and no multifocal disease. However, the disadvantage of lobectomy is that serum Tg for evaluation of postoperative disease status and RAI remnant ablation cannot be used.

There are some limitations of this study. First, this study was retrospective in nature. Second, there may have been a selection bias because the data were collected at a single tertiary institution and do not represent the entire patient population. Third, the study population included only 94 pediatric patients with a relatively short follow-up period (148.6 ± 81.6 months, range 60–452 months). Fourth, the 30-year data do not reflect the changes in the indication of surgical extent during this period. Finally, 88 (93.5%) patients were aged >10 years and only 6 (6.4%) patients were aged <10 years; therefore, it is unlikely that the results can be generalized to the entire pediatric population.

However, the strength of this study was the follow-up of every patient and the use of a standardized laboratory and imaging protocol at a single institution. Furthermore, to the best of our knowledge, only a few studies have reported the long-term prognosis of pediatric DTC.

## Conclusions

According to the ATA management guidelines for children, BTT remains the treatment of choice for pediatric patients with DTC. Decision making regarding the surgical approach and the extent of surgery should strike a balance between the completeness of tumor removal and the quality of life of the patients. The results of this study show that comparable surgical outcomes may be achieved with lobectomy, and lobectomy may, hence, be considered for patients with limited disease, such as those with tumor size <2 cm, no suspicious lymph nodes, intrathyroidal lesion, and no multifocal disease. Further studies are required to determine whether lobectomy is useful in clinical practice for pediatric patients with DTC.
